# Supplementary figures and images for: CDMPred: a tool for predicting cancer driver missense mutations with high-quality passenger mutations
Source: PeerJ. 2024 Sep 6;12:e17991. doi: 10.7717/peerj.17991 (PMC11382650; doi:10.7717/peerj.17991)

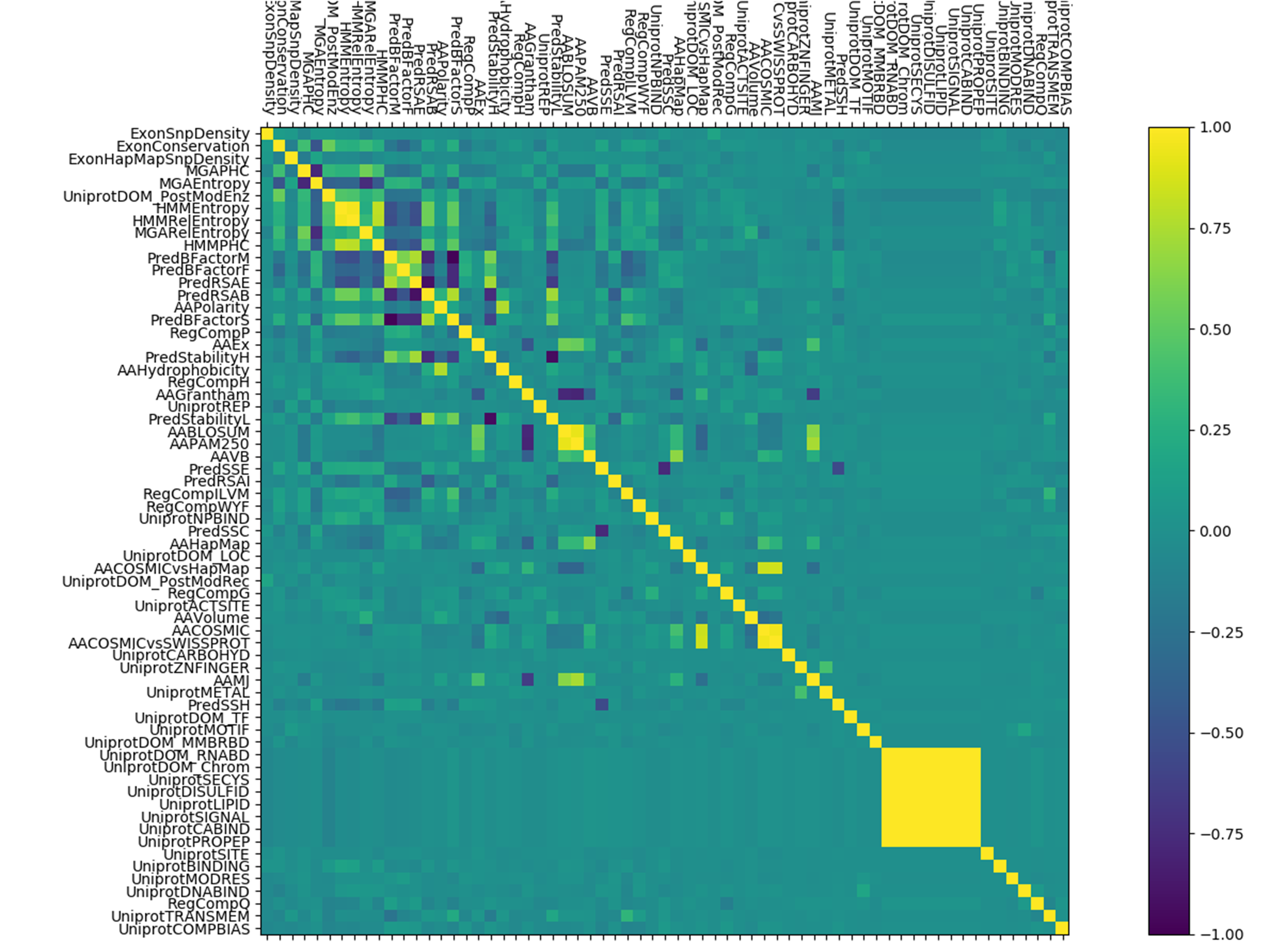

Supplement: Figure S1 [file peerj-12-17991-s001.png]
