# Supplementary material for: CDMPred: a tool for predicting cancer driver missense mutations with high-quality passenger mutations
Source: PeerJ. 2024 Sep 6;12:e17991. doi: 10.7717/peerj.17991 (PMC11382650; doi:10.7717/peerj.17991)
Supplement: Table S1 [file peerj-12-17991-s002.docx]

**Table S1. Summary of mutation datasets used for feature analysis**

| **Type** | **Mutation** | **Source** | **Description** |
| --- | --- | --- | --- |
| Negative | 1634(1548) | dbCPM v1.1 | The database of cancer passenger mutations |
|  | 380351(373143) | CHASM v3.1 | All the negative samples used in CHASM v3.1 training set |
|  | 39959(37016) | FATHMM cancer | All polymorphisms in humsavar used as passenger mutations in FATHMM cancer training set |
| Positive | 1248(1191) | DoCM v3.2 | The database of curated driver mutations |

*Notes:* FATHMM, the Functional Analysis Through Hidden Markov Model; CHASM, Cancer-Specific High-throughput Annotation of Somatic Mutations. The data in parentheses excluded mutations failed to obtain feature representation.
