# Supplementary material for: CDMPred: a tool for predicting cancer driver missense mutations with high-quality passenger mutations
Source: PeerJ. 2024 Sep 6;12:e17991. doi: 10.7717/peerj.17991 (PMC11382650; doi:10.7717/peerj.17991)
Supplement: Table S2 [file peerj-12-17991-s003.docx]

**Table S2. Definition of 65 features**

| **Category** | **Name** | **Definition** |
| --- | --- | --- |
| Protein physicochemical properties | AAVolume | The change in residue volume resulting from the replacement (in units of cubic Angstroms) |
|  | AAPolarity | Polarity change from reference to mutation amino acid residue |
|  | AAEx | Amino acid substitution score from the EX matrix. |
|  | AAVB | Amino acid substitution score from the VB (Venkatarajan and Braun) matrix |
|  | AAMJ | Amino acid substitution score from the Miyazawa-Jernigan contact energy matrix |
|  | AABLOSUM | Amino acid substitution score from the BLOSUM 62 matrix |
|  | AAPAM250 | Amino acid substitution score from the PAM250 matrix. |
|  | AAGrantham | The Grantham distance from reference to mutation amino acid residue. |
|  | AAHapMap | Ln(frequency) of missense change type in HapMap validated SNPs in dbSNP Build 129 |
|  | AAHydrophobicity | The change in hydrophobicity resulting from the substitution. |
|  | AACOSMIC | Ln(frequency) of missense change type (amino acid type X to amino acid type Y, e.g. ALANINE to GLYCINE) in COSMIC (release 38) |
|  | AACOSMICvsHapMap | Ln(frequency) of missense change in COSMIC (release 38) normalized by the number of times the change type was observed in HapMap validated SNPs in dbSNP Build 129 |
|  | AACOSMICvsSWISSPROT | Ln(frequency) of missense change in COSMIC (release 38) normalized by the frequency of reference amino acid residue in human proteins in SwissProt/TrEMBL |
| Multiple sequence alignment | **MGAPHC** | Calculated based on the degree of conservation of the residue, the mutation and the most probable amino acid in the column of a Multiz-46-way alignment from UCSC Human Genome Browser hg19. |
|  | **MGAEntropy** | The Shannon entropy calculated for the column of the Multiz-46-way alignment, corresponding to the location of the mutation. |
|  | MGARelEntropy | Kullback-Leibler divergence calculated for the column of Multiz-46-way alignment (corresponding to the location of the mutation) and that of a background distribution of amino acid residues computed from a large sample of multiple sequence alignments. |
|  | HMMPHC | Calculated based on the degree of conservation of the residue, the mutation and the most probable amino acid in a match state of a hidden Markov model built with SAM-T2K software. |
|  | HMMEntropy | The Shannon entropy calculated for the column of the SAM-T2K multiple sequence alignment, corresponding to the location of the mutation. |
|  | HMMRelEntropy | Kullback-Leibler Divergence calculated for the column of the SAM-T2K multiple sequence alignment (corresponding to the location of the mutation) and that of a background distribution of amino acid residues computed from a large sample of multiple sequence alignments. |
|  | **ExonConservation** | The conservation score for the entire exon calculated from a 46-species phylogenetic alignment using the UCSC Genome Browser (hg19). Scores are given for windows of nucleotides. We retrieve the scores for each region that overlaps the exon in which the base substitution occurred and calculated a weighted average of the conservation scores where the weight is the number of bases with a particular score. |
| Exon features | **ExonSnpDensity** | The number of SNPs in the exon where the mutation is located divided by the length of the exon. |
|  | **ExonHapMapSnpDensity** | The number of HapMap verified SNPs (dbSNP build 131) in the exon where the mutation is located divided by the length of the exon. |
| protein local structure | PredBFactorS | These features consist of the probability that the wild type residue backbone is stiff. |
|  | PredBFactorM | These features consist of the probability that the wild type residue backbone is intermediate. |
|  | PredBFactorF | These features consist of the probability that the wild type residue backbone is flexible. |
|  | PredStabilityL | These features consist of the probability that the wild stability type residue contributes to overall protein stability in a manner that is highly destabilizing, Stability estimates for the neural network training data were calculated using the FoldX force field |
|  | PredStabilityH | These features consist of the probability that the wild stability type residue contributes to overall protein stability in a manner that is highly stabilizing, Stability estimates for the neural network training data were calculated using the FoldX force field |
|  | PredSSE | These features consist of the probability that the secondary structure of the region in which the wild type residue exists is strand. |
|  | PredSSC | These features consist of the probability that the secondary structure of the region in which the wild type residue exists is loop. |
|  | PredSSH | These features consist of the probability that the secondary structure of the region in which the wild type residue exists is helix. |
|  | PredRSAB | These features consist of the probability of the wild type accessibility residue being buried. |
|  | PredRSAI | These features consist of the probability of the wild type accessibility residue being intermediate. |
|  | PredRSAE | These features consist of the probability of the wild type accessibility residue being exposed. |
| Regional composition | RegCompP | Proportion of Prolines in a 15-amino-acid-residue window centered on the reference/mutation amino acid position. |
|  | RegCompH | Proportion of Histidines in a 15-amino-acid-residue window centered on the reference/mutation amino acid position. |
|  | RegCompG | Proportion of Glycines in a 15-amino-acid-residue window centered on the reference/mutation amino acid position. |
|  | RegCompQ | Proportion of Glutamines in a 15-amino-acid-residue window centered on the reference/mutation amino acid position. |
|  | RegCompILVM | Proportion of Isoleucines, Leucines, Valines, and Methionines in a 15-amino-acid-residue window centered on the reference/mutation amino acid position. |
|  | RegCompWYF | Proportion of Tryptophans, Tyrosines, and Phenylalanines in a 15-amino-acid-residue window centered on the reference/mutation amino acid position. |
| UniProt | **UniprotCARBOHYD** | Carbohydrate binding site. |
|  | UniprotZNFINGER | Site in a zinc finger. |
|  | UniprotACTSITE | Sites involved in enzymatic activity. |
|  | **UniprotMETAL** | Metal binding site. |
|  | **UniprotREP** | Repeat region. |
|  | UniprotMOTIF | Site of known functional motif. |
|  | UniprotSECYS | Site of a selenocystein. |
|  | UniprotDISULFID | Site of disulfide bond. |
|  | UniprotLIPID | Lipid binding site. |
|  | UniprotSIGNAL | Site of localization signal (protein targeted to secretory pathway or periplasm). |
|  | UniprotCABIND | Calcium binding site. |
|  | UniprotPROPEP | Site in the propeptide (cleaved in mature protein). |
|  | UniprotSITE | An interesting amino acid site in the protein sequence. |
|  | UniprotBINDING | Binding sites. |
|  | UniprotMODRES | Site of modified residue. |
|  | UniprotDNABIND | DNA binding site. |
|  | UniprotNPBIND | Nucleotide phosphatebinding region. |
|  | UniprotTRANSMEM | Transmembrane region. |
|  | UniprotCOMPBIAS | Compositionally biased region. |
|  | **UniprotDOM_PostModEnz** | Site in an enzymatic domain responsible for any kind of post-translational modification |
|  | UniprotDOM_PostModRec | Site in a domain that recognizes a posttranslationally modified residue |
|  | UniprotDOM_LOC | Site in a domain that determines correct cellular localization of a protein. |
|  | UniprotDOM_TF | Site in a transcription factor doman. |
|  | **UniprotDOM_MMBRBD** | Site in a domain that binds to the cell membrane. |
|  | UniprotDOM_RNABD | Site in an RNA binding domain. |
|  | UniprotDOM_Chrom | Site in a domain involved in chromatin structure remodeling. |

Note: these features are also included in CanDrA ([Mao, et al., 2013b](#_ENREF_28)) and the bolded features are final top 10 features used in CDMPred.
