# Supplementary material for: CDMPred: a tool for predicting cancer driver missense mutations with high-quality passenger mutations
Source: PeerJ. 2024 Sep 6;12:e17991. doi: 10.7717/peerj.17991 (PMC11382650; doi:10.7717/peerj.17991)
Supplement: Table S3 [file peerj-12-17991-s004.docx]

**Table S3** Comparison of performance on the training set with 10-fold cross validation between CDMPred and models trained on class labels using random permutation

| **Method** | **AUC** |
| --- | --- |
| CDMPred | 0.826 |
| CDMPred_random | 0.500±0.017 (***) |

significance levels: *** p-value < 0.001
