# Supplementary material for: CDMPred: a tool for predicting cancer driver missense mutations with high-quality passenger mutations
Source: PeerJ. 2024 Sep 6;12:e17991. doi: 10.7717/peerj.17991 (PMC11382650; doi:10.7717/peerj.17991)
Supplement: Table S4 [file peerj-12-17991-s005.docx]

**Table S4** Comparison of performance on the independent test set between CDMPred and other methods designed to predict single nucleotide driver variants in cancer using the Delong test

| **Method** | **P-value** |
| --- | --- |
| Cscape-somatic | <0.0001 |
| TransFIC | <0.0001 |
| FATHMM | <0.0001 |
| CanDrA | <0.0001 |
| CHASM_plus | <0.0001 |
| CHASM | <0.0001 |
