# Supplementary material for: CDMPred: a tool for predicting cancer driver missense mutations with high-quality passenger mutations
Source: PeerJ. 2024 Sep 6;12:e17991. doi: 10.7717/peerj.17991 (PMC11382650; doi:10.7717/peerj.17991)
Supplement: Table S5 [file peerj-12-17991-s006.docx]

**Table S5** Comparison of performance on the independent test set between CDMPred and other general-purpose predictors using the Delong test

| **Method** | **P-value** |
| --- | --- |
| MetaSVM | <0.0001 |
| MetaLR | <0.0001 |
| M-CAP | <0.0001 |
| REVEL | 0.00014 |
| DANN | <0.0001 |
| CADD | 0.09667 |
| SIFT | <0.0001 |
| PolyPhen2 | <0.0001 |
| MutationAssessor | <0.0001 |
| MVP | <0.0001 |
